# Supplementary figures and images for: Strategies for improved rhamnolipid production by Pseudomonas aeruginosa PA1
Source: PeerJ. 2016 May 24;4:e2078. doi: 10.7717/peerj.2078 (PMC4888285; doi:10.7717/peerj.2078)

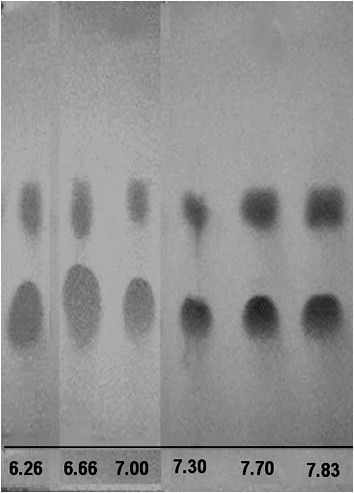

Supplement: Supplemental Information 1 — In the image shows the chromatographic migration profiles of rhamnolipids synthesized by P. aeruginosa in culture media with different pH values. The horizontal line indicates the point of application of the samples and in the base of the chromatographic plate are recorded the values of pH of the culture media where rhamnolipids were produced. The chromatography was carried out in silica-gel-coated aluminum sheets (Macherey-Nagel®) using CHCl3:CH3OH:CH3COOH (65:15:2) as the eluent. [file peerj-04-2078-s002.jpg]
